# Baseline wander and power-line interference elimination of ECG signals using efficient signal-piloted filtering

**DOI:** 10.1049/htl.2019.0116

**Published:** 2020-09-03

**Authors:** Saeed Mian Qaisar

**Affiliations:** Department of Electrical and Computer Engineering, EFfat University, Jeddah, 21478, Kingdom of Saudi Arabia

**Keywords:** electrocardiography, medical signal processing, filtering theory, power-line interference elimination, ECG signals, electrocardiogram signals, signal variations, inferior order filters, MIT-BIH ECG database, signal-to-noise ratio, 2.18-fold compression gain, computational efficiency, analogous output SNR, baseline wander interference elimination, signal-piloted linear phase filtering tactics, eigenvalue decomposition based tactics

## Abstract

A signal-piloted linear phase filtering tactic for removing baseline wander and power-line interference from the electrocardiogram (ECG) signals is suggested. The system is capable of adjusting its parameters by following the incoming signal variations. It renders the processing of lesser samples by inferior order filters. The applicability is demonstrated by using the MIT-BIH ECG database. The precision of the approach is also studied regarding the signal-to-noise ratio (SNR). Results showed that the proposed method achieves a 2.18-fold compression gain and notable computational efficiency over conventional counterpart while securing an analogous output SNR. A comparison of the designed solution is made with the contemporary empirical mode decomposition with Kalman filtering and eigenvalue decomposition based tactics. Results show that the suggested method performs better in terms of output SNR for the studied cases.

## 1 Introduction

Power-line interference (PLI) and baseline wander (BW) are the major noise elements, present in the electrocardiogram (ECG) signals [1, 2]. The BW artefacts are introduced by respiration. These are of very low-frequency and mainly occurs between [0; 0.7] Hz [2]. The PLI is introduced because of the electromagnetic interference of the alternating supply. Depending on the power supply, the frequency of PLI is 50 Hz or 60 Hz. The involvement of these noise signals diminishes the diagnosis accuracy [3]. Therefore, removal of BW and PLI is obligatory for a precise diagnosis of the cardiac diseases [4, 5]. In this framework, numerous techniques have been presented, such as extended Kalman filter (EKF) [6], Hilbert vibration decomposition (HVD) [7], adaptive-filtering [8], and eigenvalue decomposition (EVD) [4].

Effective cardiac failure treatment can be realised with a real-time ECG signal monitoring by using the ECG wearables. The Nyquist-based signal processing governs the operation of these systems. They are time-invariant, which results in a worst-case parameterisation [9, 10]. The system computational load and processing plus transmission activities remain fixed irrespective of the ECG signal intermittence and time-variations. Therefore, it can augment the computational complexity and power consumption. The signal-piloted ECG acquisition approaches have been suggested to compensate for these shortfalls. These are based on level-crossing sampling (LCS) [11, 12].

## 2 Proposed method

**Fig. 1:**
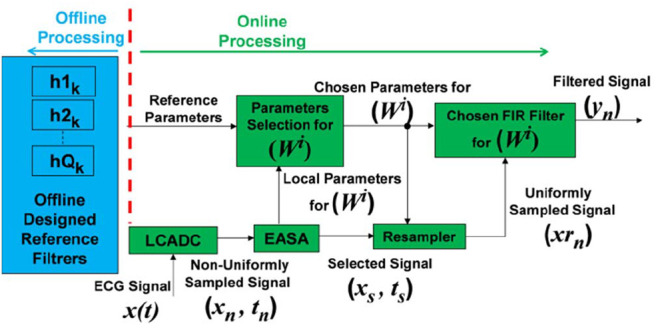
Block diagram of the proposed system

This work aims to contribute to the development of novel computationally efficient ECG diagnosis systems in a wireless sensing and cloud-based analysis environment. The realisation is achieved by using an intelligent assembly of the level-crossing A/D converters (LCADCs), the enhanced activity selection algorithm (EASA), and adaptive-rate filtering. It significantly lessens the activity of the post BW and PLI denoising module by only treating the pertinent information with adjustable order FIR filters [9, 10]. The designed system principle is shown in Fig. 1.

The MIT-BIH Arrhythmia database is employed to study the system performance [13]. Each channel is acquired with an 11-bit resolution ADC at an acquisition frequency of 360 Hz. A set of 5, 30.1-min duration recordings are used. A BW signal of 30.1-min duration recording is also employed from the MIT-BIH noise stress test database [13]. The PLI is modelled as sinusoid of 60 Hz frequency. The intended ECG recordings are denoised by a digital filter to diminish the impact of BW and PLI [4]. The noisy signal $x\left(t \right)$ is generated by adding BW noise $n_{{\rm BW}}$ and PLI noise $n_{{\rm PLI}}$ in a clean ECG signal $y\left(t \right)$. The process can be expressed as

(1) $$x\left(t \right)= y\left(t \right)+ n_{{\rm BW}} + n_{{\rm PLI}}\eqno\lpar 1\rpar $$

The band-limited, $\left[{Fc_{\min } = 0.5\semicolon \; \; \, Fc_{\max } = 60} \right]\, {\rm Hz}$, ECG signal $x\left(t \right)$ is acquired with an LCADC. It employs 21 thresholds, symmetrically and uniformly placed within the LCADC dynamics $\Delta V = 2\, {\rm V}$. It results in *M* = 4.39-bit and *q =* 0.0952 V [11]. The choice of the number of thresholds is made based on the intended application. For LCADC, the sampling is triggered only when $x\left(t \right)$ traverse one of the prefixed thresholds. Samples are irregularly spaced in time and the sampling density is piloted by the $x\left(t \right)$ variations. Equation (2) represents the sampling instants of the level-crossing samples. Where $t_n$ is the current instant, $t_{n - 1}$ is the precedent one and ${\rm d}t_{n - 1}$ is the time step among them

(2) $$t_n = t_{n - 1} + {\rm d}t_{n - 1}\eqno\lpar 2\rpar $$

The conversion process of an LCADC is dual as compared to the classical counterparts. The sample amplitudes are ideally known for an ideal LCADC, and the sampling instants are quantified according to the resolution of the timer circuit and its frequency of operation $F_{{\rm Timer}}$. Unlike the traditional approach, the signal-to-noise ratio (SNR) of LCADC does not base on resolution *M* and quantum *q*. However, it is a function of the $T_{{\rm Timer}} = \left({1/F_{{\rm Timer}}} \right)$ [11].

**Fig. 2:**
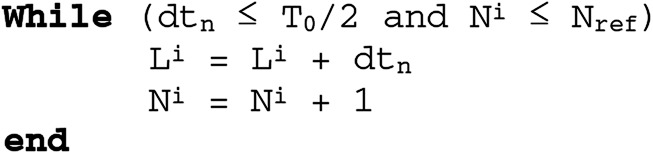
Enhanced activity selection algorithm

In practice, digital signal processing is conducted on the finite-length segments. Therefore, the EASA is used to segment the LCADC output. It segments the pertinent signal information by using the sampling process non-uniformity. The principle is clear from the following algorithm (see Fig. 2).

Here $T_0$ is the fundamental period of $x\left(t \right)$. $T_0$ with ${\rm d}t_n$ identifies the activity [10]. The condition on ${\rm d}t_n$ allows to respect the Nyquist criterion for the lowest frequency component $Fc_{\min }$ of $x\left(t \right)$. $L^i$ is the length of the *i*th selected segment $W^i$. $N^i$ is the number of samples exists in $W^i$. $N_{{\rm ref}}$ is the superior bound on $N^i$ and its selection depends on the system parameters. In this case, $N_{{\rm ref}} = 2048$ is selected. At the beginning of each iteration, ‘*i*’ is incremented and $N^i$ and $L^i$ are initialised to zero.

The traditional windowing functions [10] do not give any interesting features of the EASA. Only the appropriate signal information is chosen, and the length of each segment is modified by following local characteristics of the segmented signal. The sampling frequency for $W^i$ can be measured as $Fs^i = \left({N^i/L^i} \right)$. The $W^i$ is uniformly resampled using the simplified linear interpolator (SLI) [10] to take advantage of the existing classical processing techniques. Compared to the resampled signal modifies compared to the non-uniform signal. This variation is a function of *M*, *q* and the interpolator [14]. The superior error limit per resampled observation is $\left({q/2} \right)$ for SLI [14].

**Fig. 3:**
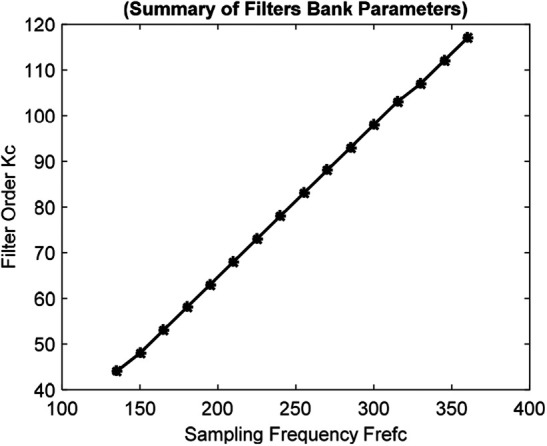
Summary of filters bank parameters

A finite impulse response (FIR) filters bank is offline designed for the proficiently online diminishing of the BW and the PLI from the ECG signals. The useful frequency range of the ECG signal is between [0.5; 45] Hz [4]. A band-pass filters bank is offline designed for the cut-off frequencies of $\left[{Fc_L = 0.5\semicolon\; Fc_H = 45} \right]{\rm Hz}$. It allows focusing on the ECG band of interest while attenuating the BW and PLI noise [4]. The filters bank is designed for a set of sampling frequencies, *Fref*, between $Fs_{\min } = 135\, {\rm Hz} \gt 2\comma \; Fc_{\max }\, {\rm to}\, F_r = 360\, {\rm Hz}$. In this case, Δ = 15 Hz is chosen. It realises a bank of *Q* = 16 band-pass FIR filters. Here, $\Delta = \left({F_r - Fs_{\min }/Q - 1} \right)$. The sampling frequencies and orders of the reference filters are summarised in Fig. 3. It shows that for the selected specification, the lowest 44th-order filter in the bank is obtained for $Fref_1 = Fs_{\min } = 135\, {\rm Hz}$, and the highest 117th-order filter in the bank is obtained for $Fref_{32} = F_r = 360\, {\rm Hz}$.

**Fig. 4:**
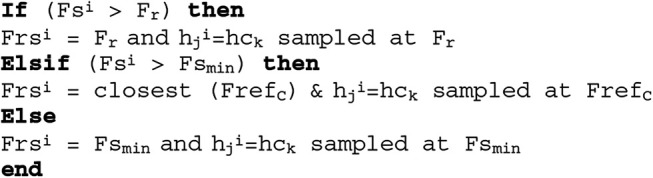
Algorithm for online choosing the resampling frequency and the denoising filter for W^i^

The EASA analyses properties of $W^i$ and uses it for modifying the post modules parameters like the resampling frequency, $Frs^i$, and the filter order, ${\bi K}_{\bi C}$. An effective filter is selected online from the reference set for each $W^i$. Let $h_{c_k}$ be the selected filter for $W^i$ and is sampled at $Fref_C$. This selection is made on the basis of *Fref* and $Fs^i$. For a proper filtering, the $Frs^i = Fref_C$ is chosen [10]. The method of choosing $Frs^i$ and keeping it aligned with $Fref_C$ is described in Fig. 4.

## 3 Performance evaluation

The designed system performance is evaluated in terms of compression gain, online processing efficiency, and output quality.

In the classical case, $x\left(t \right)$ is acquired at a fixed frequency $F_r$. Therefore, the count of samples, *N*, for a considered time length $L_T$ is straightforward to compute as $N = F_r \times \; L_T$. For LCADC, the sampling frequency is not unique and is piloted by variations in $x\left(t \right)$. If $N_{ED}$ is the number of samples obtained at the output of LCADC for $L_T$. Then the compression gain can be calculated as

(3) $$G_{{\rm COMP}} = \displaystyle{N \over {N_{ED}}}\eqno\lpar 3\rpar $$

The computational complexity of a classical *K* order FIR filter is known. It performs *K* additions and *K* multiplications while calculating an output sample. For *N* samples, the computational cost *C*_FIR_ can be computed as

(4) $$C_{{\rm FIR}} = \underbrace{{K \cdot N}}_{{{\rm Additions}}} + \underbrace{{K \cdot N}}_{{{\rm Multiplications}}}\eqno\lpar 4\rpar $$

For the suggested solution, the online filter selection and the selected segment resampling processes necessitate additional operations. The filter selection for $W^i$ is resolved by using the successive approximation algorithm. Therefore, it requires $\log _2\left(Q \right)$ comparisons for the worst case [10]. Here, *Q* is the length of the set *Fref*. The resampling is realised by using the *SLI*. For $W^i$, the complexity of *SLI* is $Nr^i$ additions and $Nr^i$ binary-weighted right shifts. The complexity of the binary-weighted right shift is negligible compared to the addition and multiplication processes [15]. Therefore, the complexity of proposed adaptive-rate FIR (ARFIR) method for $W^i$ can be calculated as

(5) $$C^i_{{\rm ARFIR}} = \underbrace{{K^i \cdot Nr^i + Nr^i + {\log }_2\left(Q \right)}}_{{{\rm Additions}}} + \underbrace{{K^i \cdot Nr^i}}_{{{\rm Multiplications}}}\eqno\lpar 5\rpar $$

Accuracy of the proposed solution is evaluated in terms of the SNR. The noisy signal $x_n$ is the input and the filtered signal $yf_n \simeq y_n$ is the system output. Here, $x_n$ and $y_n$ are the digital versions of $x\left(t \right)$ and $y\left(t \right)$. The ${\rm SN}{\rm R}^i$ for $W^i$ is calculated as

(6) $${\rm SNR}_{{\rm dB}}^i \; = 10 \cdot \log _{10}\left({\displaystyle{{\displaystyle{1 \over {Nr^i}}\sum\nolimits_{l = 1}^{Nr^i} {{\left({y_n} \right)}^2} } \over {\displaystyle{1 \over {Nr^i}}\sum\nolimits_{l = 1}^{Nr^i} {{\left({e_n} \right)}^2} }}} \right).\eqno\lpar 6\rpar $$

In (6), $e_n$ is the error per observation and it is calculated as the absolute difference between $y_n$ and $yf_n$. After calculating ${\rm SN}{\rm R}^i$ the overall SNR for an intended ECG recording of 30.1-min, the duration is calculated as the average ${\rm SN}{\rm R}^i\, {\rm s}$ of all selected segments.

The designed solution performance is compared with the traditional one in terms of compression gain and processing efficiency. The system precision is compared with the vital contemporary counterparts, based on the EVD [4] and the empirical mode decomposition (EMD), the wavelet transform (WT) [16] with the EKF [6]. Techniques of [6, 16] are merged as EMD-WT-EKF [4].

## 4 Simulation results

**Fig. 5:**
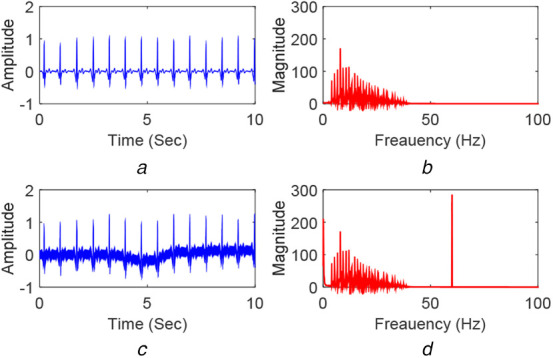
Examples of clean and noisy ECG signals and their spectrum *a* Clean ECG signal $y_n$ *b* Magnitude spectra of $y_n$ *c* Noisy ECG signal $x_n$ *d* Magnitude spectra of $x_n$

An example of the noise-free signal, $y\left(t \right)$, is shown in Fig. 5*a*. It is a segment of record number 100. Its magnitude spectra are shown in Fig. 5*b*. The noisy signal $x\left(t \right)$ with 0 dB SNR is shown in Fig. 5*c*. The magnitude spectrum of the noisy signal is shown in Fig. 5*d*. The presence of BW and PLI noise is observable by comparing the time-domain plots of Figs. 5*a* and *c*. In Fig. 5*d*, additional spikes exist compared to Fig. 5*b*. These spikes occur at the low-frequency region and at 60 Hz and, respectively, confirm the presence of BW and PLI noise.

**Fig. 6:**
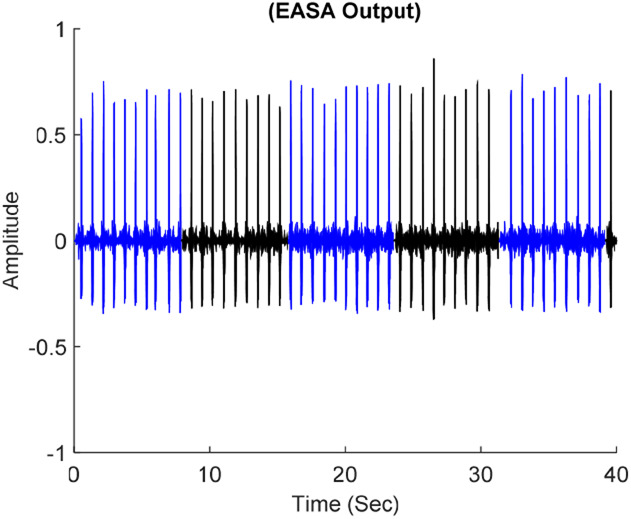
EASA output odd segments in blue colour and even segments in black colour

The first 40 s of record number 100, digitised with a 4.39-bit LCADC and afterwards segmented with the EASA is shown in Fig. 6. For employed parameters, the EASA delivers six selected segments.

The selected segments’ parameters are outlined in Table 1. It describes the attractive characteristics of the proposed approach, which are adapting $L^i$, $Fs^i$, $Frs^i$, $Nr^i$, and $K_C$ for $W^i$. $L^i$ demonstrates that how for a chosen $N_{{\rm ref}} = 2048$, the time-length of each $W^i$ is adjusted according to the temporal disparities of the incoming signal. Contrary, in the classical counterpart for $N_{{\rm ref}} = 2048$ and $F_r = 360\, {\rm Hz}$ the length of each segment is unique and equal to 5.68 s regardless of the incoming signal sporadic nature. $Nr^i$ and $K_C$ demonstrate that how the tuning of $Frs^i$ contributes to the proposed method processing gain by reducing the superfluous interpolations and denoising operations.

**Table 1:** Summary of the selected segments parameters

*W_i_*	$L^i$, s	$Fs^i$, Hz	$Fref_C$, Hz	$Frs^i$, Hz	*Nr^i^* (samples)	KC
1st	8.75	234.06	225	225	1968	73
2nd	8.72	234.86	225	225	1968	73
3rd	6.89	297.24	285	285	1963	93
4th	6.85	298.98	285	285	1952	93
5th	7.82	261.89	255	255	1994	83
6th	0.98	218.41	210	210	0206	68

These outcomes are compared with the traditional method. The complete $x\left(t \right)$ span, 30.1-min, is sampled at $F_r = 360\, {\rm Hz}$ in the conventional case. It results in 650k samples to denoise with a 117th-order FIR filter. Nevertheless, with the suggested strategy, the entire collection of acquired information points is smaller. Additionally, local filter orders, for most of the selected segments, are reduced than 117th. Compared to the classical method, it gives impressive compression gain and processing efficacy of the suggested approach.

**Fig. 7:**
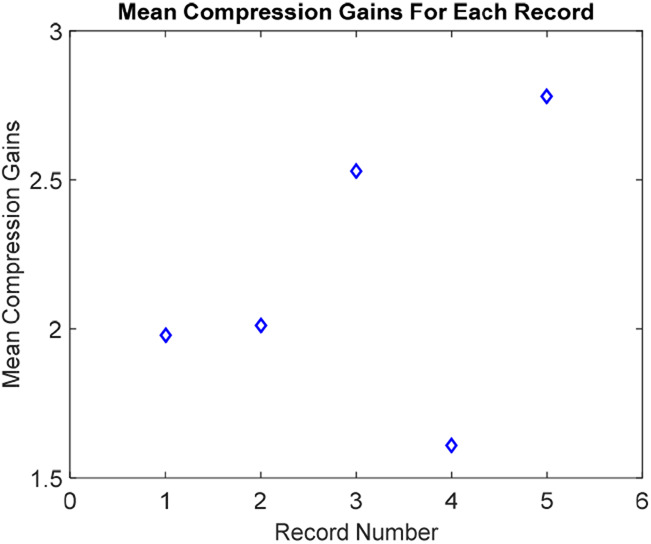
Mean compression gains for intended records

The compression gains of the suggested method over the classical counterpart are also calculated for each intended recording of 30-min duration. The findings are summarised in Fig. 7. For proper plotting, the record numbers are incremented sequentially in Fig. 7. Each record is presenting a real one from the MIT-BIH dataset. It is clear from Table 2.

**Table 2:** Look-up table for plotted and MIT-BIH records

record no. (plotted)	1	2	3	4	5
record no. (MIT-BIH)	100	101	115	116	117

Fig. 7 shows that the minimum compression gain of 1.61-fold is attained for the MIT-BIH record number 116. The maximum compression ratio of 2.78-fold is attained for the MIT-BIH record number 117. The overall mean compression gain for all intended 5-records is 2.18-fold.

**Fig. 8:**
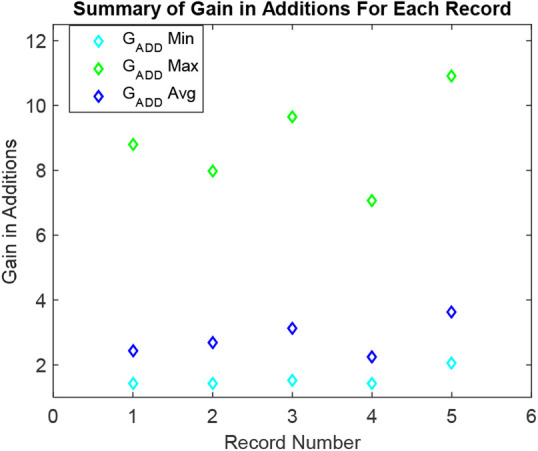
Summary of gains in additions for intended records

For considered 5-ECG records, the processing gains of the ARFIR over the classical counterpart are also computed. Firstly, the processing gains in terms of additions and multiplications are calculated for each selected segment. It resulted in the minimum and the maximum gains in additions of 1.43-fold and 8.8-fold for all selected segments of the record number 100. The average gain in additions for all selected segments of record number 100 is 2.42-fold. The minimum and the maximum gains in additions are of 1.44-fold and 7.98-fold for all selected segments of the record number 101. The average gain in additions for all selected segments of record number 101 is 2.7-fold. The minimum and the maximum gains in additions are of 1.53-fold and 9.66-fold for all selected segments of the record number 115. The average gain in additions for all selected segments of record number 115 is 3.14-fold. The minimum and the maximum gains in additions are of 1.42-fold and 7.06-fold for all selected segments of the record number 116. The average gain in additions for all selected segments of record number 116 is 2.24-fold. The minimum and the maximum gains in additions are of 2.04-fold and 10.91-fold for all selected segments of the record number 117. The average gain in additions for all selected segments of record number 117 is 3.64-fold. A summary of these findings is plotted in Fig. 8. Each record in the plot is presenting a real one from the MIT-BIH dataset. It is clear from Table 2.

**Fig. 9:**
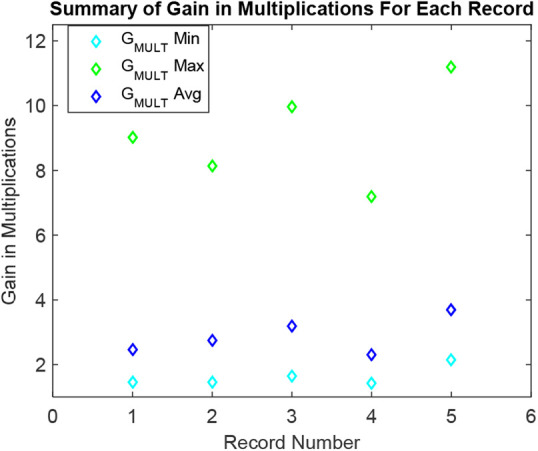
Summary of gains in multiplications for intended records

The minimum and the maximum gains in multiplications are of 1.45-fold and 9.01-fold for all selected segments of the record number 100. The average gain in multiplications for all selected segments of record number 100 is 2.45-fold. The minimum and the maximum gains in multiplications are of 1.46-fold and 8.15-fold for all selected segments of the record number 101. The average gain in multiplications for all selected segments of record number 101 is 2.75-fold. The minimum and the maximum gains in multiplications are of 1.65-fold and 9.97-fold for all selected segments of the record number 115. The average gain in multiplications for all selected segments of record number 115 is 3.20-fold. The minimum and the maximum gains in multiplications are of 1.44-fold and 7.20-fold for all selected segments of the record number 116. The average gain in multiplications for all selected segments of record number 116 is 2.30-fold. The minimum and the maximum gains in multiplications are of 2.16-fold and 11.19-fold for all selected segments of the record number 117. The average gain in multiplications for all selected segments of record number 117 is 3.70-fold. A summary of these findings is plotted in Fig. 9. Each record in the plot is presenting a real one from the MIT-BIH dataset. It is clear from Table 2.

The efficiency of the suggested approach is also compared to the traditional counterpart in terms of output SNR, and results are outlined in Table 3. It shows that the output SNR of the filtered signal obtained using the suggested signal-piloted adaptive-rate denoising is similar to the output SNR of the filtered ECG signals obtained using the traditional filtering tactic.

**Table 3:** Values of output SNR for BW + PLI noise at 0 dB SNR_in_

record number	100	101	115	116	117
SNR, dB, proposed	15.62	13.39	16.20	13.38	14.80
SNR, dB, classical approach	15.85	13.45	16.59	13.64	15.08

The performance of the suggested method in terms of output SNR is compared with the existing state-of-the-art tactics. In this study, EVD [4] and EMD-WT-EKF approaches are considered [6, 16]. The findings are outlined in Table 4. It shows that for record number 100, the suggested method secures, respectively, 4.1 and 7.32 dB superior output SNR compared to the EVD and the EMD-WT-EKF tactics. For record number 101, the suggested method secures, respectively, 1.28 and 4.54 dB superior output SNR compared to the EVD and the EMD-WT-EKF tactics. For record number 115, the suggested method secures, respectively, 4.09 and 7.34 dB superior output SNR compared to the EVD and the EMD-WT-EKF tactics. For record number 116, the suggested method secures, respectively, 3.82 and 4.23 dB superior output SNR compared to the EVD and the EMD-WT-EKF tactics. For record number 117, the suggested method secures, respectively, 3.95 and 5.65 dB superior output SNR compared to the EVD and the EMD-WT-EKF tactics.

**Table 4:** Values of output SNR for BW + PLI noise at 0 dB SNR_in_

record number	100	101	115	116	117
SNR, dB, proposed	15.62	13.39	16.20	13.38	14.80
SNR, dB, EVD [4]	11.52	10.78	12.11	9.56	10.85
SNR, dB, EMD-WT-EKF [6, 16]	8.30	8.85	8.86	9.15	9.15

## 5 Discussion

For the case of studied 5-ECG 30.1-min records, the performance of the designed solution is studied. Results have shown a notable compression and processing effectiveness of the suggested method compared to the conventional counterpart. The average processing gains of 2.83 times and 2.88 times, respectively, in terms of additions and multiplications compared to the conventional equivalent. It is attained by automatically organising the parameters of acquisition, segmentation, resampling and denoising by following the incoming signal variations. Compared to traditional counterparts, it results in a substantial computational gain of the suggested framework.

Table 3 shows that the system also secures an analogous performance in terms of the output SNR in comparison with the conventional counterpart. Additionally, Table 4 confirms that the devised method attains the higher values of output *SNR*, for all considered ECG recordings at 0-dB SNR_in_, compared to EVD and EMD-WT-EKF based solutions [4, 6, 16].

Another ability of the designed method compared to the previous ones is to introduce a real-time signal-piloted compression gain. The proposed architecture has reached an average compression gain of 2.18-fold for the studied 5-ECG records. It brings notable processing efficiency during the post-processing modules, which is clear from Figs. 8 and 9. It promises a similar factor of gain in terms of transmission and post-analysis module activities [10, 12]. The idea of embedding the signal-piloted acquisition and processing in the automatic cardiovascular diagnostic is quite novel [9, 12, 17]. The above results assure that a wise integration of this approach can also introduce a significant processing efficiency in other ECG denoising tactics such as the EVD [4], and the EKF-EMD-WT [6, 16].

## 6 Conclusion

To denoise BW and PLI from the ECG signals, novel signal-piloted acquisition and FIR filtering concepts are devised. The signal-piloted tactic allows real-time self-organisation of the system parameters. It resulted in a 2.18-fold compression gain and more than 2.8-folds gains in additions and multiplications compared to the classical counterpart. It is shown that the designed solution indeed secures comparable output SNR efficiency compared to the conventional counterpart. In addition, the underperformance of the developed approach is also demonstrated in terms of the output of the SNR over the state-of-the-art strategies. It is demonstrated that the proposed solution also secures analogous output SNR performance in comparison with the traditional counterpart. Moreover, the outperformance of the designed solution is also demonstrated over the state-of-the-art strategies in terms of the SNR performance. This ensures the advantage of incorporating the designed solution within the concurrent wireless and low power ECG implants.
